# Social networks for eHealth solutions on cloud

**DOI:** 10.3389/fgene.2013.00171

**Published:** 2013-09-03

**Authors:** Briti Deb, Satish N. Srirama

**Affiliations:** Mobile Cloud Laboratory, Institute of Computer Science, University of TartuTartu, Estonia

**Keywords:** cloud computing, eHealth, crowdsourcing, social networks, semantic web, participatory medicine, utility computing/on-demand computing

## Introduction

Traditional experimentation based healthcare solutions are constrained by limited data that can confirm or refute the initial hypothesis. Big medical data in individual Electronic Health Records, labs, imaging systems, physician notes, medical correspondence and claims, provides a resource for extracting complementary information that can enhance the data available from traditional approaches based on experimentation. Datamining algorithms are being used to analyze data to get a more insightful understanding of human health, both preventive and clinical. But despite their sophistication, they are far from flawless. One way to solve the problem is crowdsourcing citizens connected in a social network, who can provide data, get it analyzed, and consume data for preventive health insights (Swan, 2009). Several challenges come along with it, for instance: performance, scalability, speed, storage, and power, which we believe could be addressed by cloud-enabled social networks for eHealth services. Such services could be composed of many other services, for instance, user authentication, email, payroll management, calendars, tele-consultation, e-Prescribing, e-Referral, e-Reimbursement, and alerting services, aiming to change the way big medical data in social networking web sites could be used making it actionable to save lives.

This paper aims to explore the opportunities and challenges for realization of cloud-enabled social networks for eHealth solutions, by examining efforts already underway, and recommending solutions to improve it. We discuss a three-tier ecosystem to advance this key field leveraging the Cloud computing technologies. In Tier-1 is “*Build Sustainable eHealth System”* to create a foundation that facilitates secure creation, storage, exchange, and analysis of data between actors. In Tier-2 is “*Crowdsourced Social Networks for eHealth Services”* to utilize the power of crowdsourcing. In Tier-3 is “*Increasing Access to eHealth*” to minimize risk and improve patient outcome. Failure to address these issues is believed to result in inefficient use of big medical data toward preventive healthcare.

## The three-tier ehealth ecosystem on cloud

### Tier 1: build sustainable ehealth system

#### Semantic interoperability

As healthcare institutes do not strictly conform to a single commonly agreed vocabulary/standard, integrating bio-medical data using domain ontologies is far from perfect (Della Valle et al., 2005; Ulieru et al., 2006). We believe that such diverse data in terms of volume, variety, and velocity, can be attempted to be semantically integrated, shared, reused, and made accessible, by using a top-level ontology for integrating domain ontologies, semantic web standards such as RDF for describing information, SPARQL as an RDF query language, and OWL to represent knowledge.

#### Compliance/accountability

Specific compliance/accountability requirements can be enforced by laws and regulations on organizations that collect, generate or store medical data, thereby dictating a wide array of data related policies such as, retention time, deletion process, recovery plans, and sharing policy. Laws such as the Health Insurance Portability and Accountability Act (HIPAA) in the US are already in force and complied with by organizations like *PatientsLikeMe.com*. The Federal Risk and Authorization Management Program (FedRAMP) is another law in the US enacted to assess and authorize cloud products and services. The dispersed geographic location of cloud providers such as *Amazon.com* opens the possibility of breach of compliance, which could be addressed by *Portable Consent*, and Institutional Review Board could be enacted to monitor, approve, or prevent the use of medical data on the cloud.

#### Security and privacy

Hosting data in the cloud poses privacy concerns because the service provider may access, accidentally or deliberately alter, or even delete information. Methods to obfuscate individual identity attributes such as Zero-knowledge Technology or Privacy Enhancing Technologies are currently not used in a pervasive manner (Bertino et al., 2009) due to lack of granularity in the Access Control List, creating privacy risks. To mitigate some of the security risks such as sensitive data access, data segregation, bug exploitation, recovery, accountability, and activity by malicious insiders, solutions are being researched such as cryptography, public key infrastructure (PKI), standardisation of APIs, and virtual machine security.

#### Legislative influence

As the Cloud poses a challenge on “possession,” “custody,” and “ownership” of data, Terms of Service (TOS) agreements become vital to clarify the different rights to be assigned to different roles. The TOS must also specify procedures to follow in the event of an end of provider-customer relationship, a merger of one provider with another, bankruptcy, and insolvency. An open challenge is how to ascertain legal jurisdiction if disputes arise for geographically dispersed data. Patient Advocacy Groups could play a role in influencing advisory panels toward adopting better laws to protect providers and consumers.

#### Revenue/financial model

Crowdsourced eHealth social networks are mostly free of subscription fees, advertising, banner ads or popups. Sale of anonymized data, clinical trial awareness programs, and market research surveys constitute a major part of revenue. In future, revenue model could increasingly include health insurers, such as the already implemented *Health Savings Account* in US.

#### Reputation/credibility, quality control, and transparency

The success of safety-critical systems depends largely on the reputation/credibility they enjoy in market. Several non-technical challenges arises from the change in the IT department's role from provider to consultant (Khajeh-Hosseini et al., 2010), resulting in an increased risk to customer satisfaction, job quality, and job satisfaction, tensions between the expectations of different groups, questioning the long term organizational impact of Cloud migration on reliability, scalability, and cost effectiveness.

### Tier 2: crowdsourced social networks for ehealth services

Personalized preventive health maintenance comes against the backdrop of several challenges such as difficulty in understanding the causations of complex diseases due to an incomplete understanding of the complexities of biology, the high cost of healthcare, an aging population, and a physician shortage. One solution is to use social networks as a platform to facilitate the participation of millions of users in the crowd to realize the 4P's of medicine—preventive, personalized, predictive, and participatory. Several eHealth social networks have appeared, namely, *patientslikeme.com, hellohealth.com, medhelppc.org, curetogether.com, dailystrength.org, FacetoFaceHealth.com, 23andMe.com, Genomera.com, QuantifiedSelf.com, DIYgenomics.org*, providing a platform for people in the crowd to compare their conditions with other individuals, and identifying areas for further scientific research on their own before clinical symptoms appear. Studies have shown typical challenges for a crowdsourced system (Doan et al., 2011) such as (a) recruitment, retention, and evaluation of users, (b) merging/combining contribution of users, (c) managing quality of contribution of users, (d) managing query semantics, query execution, and query optimization, and (e) improving user interfaces.

In addition to identifying potential pre-clinical symptoms, datamining algorithms can be applied to the discussion forums provided by the eHealth social networks to identify epidemiological patterns such as (i) patient behavior in response to a safety event, (ii) efficacy and side-effects of drugs that have not shown up in trials, thereby helping to reduce time spent in clinical trial, (iii) monitoring and participating in real-world natural experiments, (iv) anonymously sharing treatment, symptom, progression and outcome data.

However, performance and adaptability of eHealth social networks face challenge due to complexities in big data handling, such as variety, velocity, volume, distribution, synchronization, fault recovery, etc. To address the challenge of distributing data and computation loads over multiple processing units, largely three main directions have being studied: (a) parallel computing frameworks such as MapReduce, Iterative MapReduce, and Bulk Synchronous Parallel (BSP), (b) Graphics Processing Units, and (c) Message Passing Interfaces.

In the MapReduce model, parallelism is achieved by executing Map and Reduce tasks concurrently. To achieve fault tolerance, data is replicated and failed tasks are re-executed. The efficiency and scalability of algorithms on the Cloud can be affected by the characteristics of an algorithm, necessitating a classification for algorithms (Srirama et al., 2012). As the MapReduce model is most suitable for embarrassingly parallel tasks, i.e., parallel tasks having little or no dependency between them, serious issues arise when working with graph problems in social networks due to factors such as (a) long “*start up”* and “*clean up”* times, (b) no way to keep important data in memory between MapReduce job executions, and (c) reading of all data from file system (HDFS) after each iteration and writing back there at the end. Three main directions are currently being pursued to address the challenges of graph processing in parallel environment: (i) restructuring algorithms for the non-iterative MapReduce version, (ii) restructuring non-iterative MapReduce algorithms into iterative MapReduce versions using alternative MapReduce frameworks (Twister, HaLoop, Spark), giving up advantages of the MapReduce model such as Fault tolerance and running multiple concurrent reduce tasks, and (iii) alternative distributed computing models such as BSP (Pregel, Hama, Giraph).

### Tier 3: increasing access to ehealth

Several challenges limit access to eHealth. One such is the workflow challenge, arising for several reasons such as the inefficiency of current processes and the dependency on paper to store data. It is envisioned that in future, a physician would enter patient data in an electronic scheduling system on the Cloud, which would be processed by some workflow to automatically determine the most appropriate test, and the patient directly notified of the possible options.

Semantically integrating diverse patients medical records, census data, and environmental samplings, and managing scalability and load balancing, are some of other major challenges while analyzing big data. One approach to addressing these is the use of virtualization technology, which allows applications to be easily migrated from one physical server to another, resulting in improved reliability, scalability, business continuity, load balancing, hardware maintenance, disaster recovery, and better utilization of processors and memory.

Yet another challenge to increasing access to healthcare is providing ubiquitous healthcare monitoring. Traditionally, patients were “treated” only in hospital/clinic, which is expected to change in future, as ubiquitous gadgets such as mobile phones are now being increasingly being used to track patients and keep them compliant. Mobile cloud computing is expected to arise as a prominent domain, seeking to bring the massive advantages of the Cloud to resource constrained smartphones, by following either the *delegation model* or *code offloading* model (Flores and Srirama, 2013). In the *delegation model*, a mobile phone consumes services from multiple clouds by following their Web API, whereas, in the *code offloading* model, a mobile application is partitioned and analyzed so that the most computationally expensive operations at code level can be identified and offloaded to the Cloud for remote processing.

## Discussion and conclusions

In this paper, we briefly analyzed the opportunities and challenges for realization of cloud-enabled social networks for eHealth solutions, and proposed a three-tier ecosystem to improve it. Four main actors can be identified: service providers (genomic counselors, biomedical researchers), remedy providers (eHealth social networks providing computing and storage), health professionals, and data provider/consumers. The challenges can be summarized into two main groups. First, technical challenges such as resource exhaustion attributed to the ever increasing demand of the Cloud resources, data transfer bottlenecks attributed to the limited network bandwidth, unpredictability of Cloud performance attributed to the inability of Cloud consumers to govern the virtual architecture owned by Cloud providers, data lock-in attributed to the discontinuity of Cloud-based eHealth services, compounded by the problem of semantic interoperability when migrating the data to another Cloud, and limitations of the non-iterative MapReduce model, particularly in scalable graph processing. Second, non-technical challenges arising from the change in the IT department's role from provider to consultant, affecting customer satisfaction and overall service quality, calling for stringent quality control and transparency measures. To address these issues, we proposed a three-tier eHealth ecosystem. In future, we propose to: (i) investigate the use of Parallel R packages to leverage multi-processor systems to speed computations with big data by explicit parallelism, implicit parallelism, and implementing map-reduce for Hadoop; (ii) develop novel algorithms for parallel classification and parallel search; and (iii) develop a novel framework for semantic integration of biological data in social networks leveraging the Cloud. We believe that a combined strategy consisting of semantic, algorithmic, and computational approaches would be useful to solve many problems in eHealth social networks on the Cloud. Biological research would benefit as researchers would be able to analyze massive amounts of complex data much more quickly, and generate hypotheses faster. Finally, the authors believe that research in that direction could enhance the scale and scope of experiments that are possible, resulting in an exponential growth in knowledge, similar to the exponential growth in data that we see today.
